# We Trust You! A Multilevel-Multireferent Model Based on Organizational Trust to Explain Performance

**DOI:** 10.3390/ijerph18084241

**Published:** 2021-04-16

**Authors:** Marisa Salanova, Hedy Acosta-Antognoni, Susana Llorens, Pascale Le Blanc

**Affiliations:** 1 WANT Research Team, Universitat Jaume I, 12071 Castelló de la Plana, Spain; llorgum@uji.es; 2 Faculty of Psychology, Universidad of Talca, 3460000 Talca, Chile; hacosta@utalca.cl; 3 Human Performance Management Group, Eindhoven University of Technology, 5612 AZ Eindhoven, The Netherlands; P.M.Le.Blanc@tue.nl

**Keywords:** healthy organizational practices, team resources, vertical trust, horizontal trust, performance

## Abstract

This study tests organizational trust as the psychosocial mechanism that explains how healthy organizational practices and team resources predict multilevel performance in organizations and teams, respectively. In our methodology, we collect data in a sample of 890 employees from 177 teams and their immediate supervisors from 31 Spanish companies. Our results from the multilevel analysis show two independent processes predicting organizational performance (return on assets, ROA) and performance ratings by immediate supervisors, operating at the organizational and team levels, respectively. We have found evidence for a theoretical and functional quasi-isomorphism. First, based on social exchange theory, we found evidence for our prediction that when organizations implement healthy practices and teams provide resources, employees trust their top managers (vertical trust) and coworkers (horizontal trust) and try to reciprocate these benefits by improving their performance. Second, (relationships among) constructs are similar at different levels of analysis, which may inform HRM officers and managers about which type of practices and resources can help to enhance trust and improve performance in organizations. The present study contributes to the scarce research on the role of trust at collective (i.e., organizational and team) levels as a psychological mechanism that explains how organizational practices and team resources are linked to organizational performance.

## 1. Introduction

Organizational trust was defined by Mayer et al. [1] as “the willingness of one party to be vulnerable to the actions of another party based on the expectation that the other party will perform a particular action important to the trustor, irrespective of the ability to monitor or control the other party” (p. 712). This definition of trust is based on expectations from both employees and organizations, and it is especially important currently because of social and economic turbulence and crisis, such as, for example, the Covid-19 pandemic. In that sense, recently, Falcone et al. [2], in a sample of 4260 Italian citizens, showed that organizational trust (i.e., in public institutions) was a relevant psychological mechanism to deal with the pandemic.

Moreover, research in management and social sciences [3,4] has shown that organizational trust can be considered a source of competitive advantage and a prerequisite for the efficient functioning of organizations and HRM [5]. Moreover, trust within organizations is critical to the performance and wellbeing of their employees [6,7], and it may foster innovative and prosocial behaviors that help to create financial advantages as well [8,9].

The way organizational trust develops has been explained by different theories, such as the social exchange theory [10]. When employees trust their leaders/coworkers because they got benefits, such as work–family programs, or experience a supportive social climate, they probably will interpret their actions in a more favorable way, and consequently, will feel the need for reciprocity. They will be more willing to accept vulnerability to leaders/coworkers by investing their energy and resources to compensate for these benefits by focusing on what they must do (for example, delivering an excellent job performance).

Furthermore, trust could be understood as a psychological mechanism that mediates between organizational resources and practices (such as work–family integration, health promotion programs, time flexibility) and organizational effectiveness and performance [11,12,13,14,15]. However, there is a knowledge gap on the role of trust at different levels of analysis (i.e., individuals, teams, and organizations). For example, as proposed by Cremer et al. [16], future research should investigate how trustworthiness perceptions of each individual employee at different levels of the organization (team, departments, etc.) affect performance from higher to lower levels in the hierarchy. Fulmer and Gelfand [3] conducted a systematic review showing that research on organizational trust has not explored the collective level, i.e., considering aggregated perceptions of employee trust. They also claim that there is still a lack of evidence about how to enhance trust at multiple levels within organizations, and about the relationship between organizational and team trust and different business outcomes, such as performance. In another recent systematic review about vertical trust and performance on a sample of 75 studies, Guinot and Chiva [17] concluded that “future studies should examine trust across different referents and levels of analysis to discover unique effects on performance” (p. 218). Organizations are essentially multilevel systems, and trust operates at different levels (i.e., individual, team, and organizational levels). Therefore, to pay attention to different levels is a theoretical and empirical imperative [18], particularly the study of trust at higher (collective) levels, such as teams and whole organizations. The main novelty of this study is that we examine the antecedents and consequences of trust at two different collective levels (i.e., team and organization) to test whether similar psychological mechanisms operate at both levels of analysis. In this way, we propose trust as an explanatory mechanism in the relationship of organizational practices and team resources with performance. The research question is twofold: (1) Can trust be considered a psychological mechanism underlying the relationships between healthy organizational practices and team resources with organizational and team performance, respectively? (2) Do healthy organizational practices and trust have cross-level effects on team performance?

## 2. Theories and Hypotheses

Positive expectations about trustworthiness and the willingness to accept vulnerability are two important dimensions of organizational trust [3]. This vulnerability is implicit in traditional definitions of trust, such as the one by Mayer et al. [1]. Rousseau et al. [19] (p. 395) also defined trust as a “psychological state comprising the intention to accept vulnerability based upon positive expectations of the intentions or behavior of another”. Employees with high levels of organizational trust are willing to rely on the company, despite the implicit risk that it will not fulfill its obligations [20]. Organizational trust results from the alignment between the organizations’ systems, structures, rewards, and relevant company objectives. In this regard, Creed and Miles [21] pointed out that Human Resources Practices that yield a perception of common goals and provide common resources should affect the perception of trust. Thus, practices implemented, and resources provided by organizations at different levels (i.e., organization and teams) are relevant in developing trust and obtaining positive outcomes, such as good performance. In the current study, we differentiate between levels and referents of organizational trust, in line with the multilevel-multireferent framework of Fulmer and Gelfand [3]. For example, trust can be found at the individual and at higher levels (i.e., team and organization), where the emergence of trust perceptions by members of the unit is important. The concept of the referent addresses the object of trust, such as trust in top management, trust in immediate supervisors, trust in coworkers, etc. Additionally, we consider performance indicators at two different levels of analysis: Organizational performance, as indicated through return on assets (ROA), and team performance as assessed by the immediate team supervisor. Even more, we used a multilevel-multireferent framework by considering two collective levels of analyses (i.e., organizations and teams) and two referents (i.e., top managers and teammates) simultaneously. Legood et al. [22] stressed the importance of looking at multiple referents of organizational trust simultaneously. This multidimensional point of view enables us to understand the different dynamics of trust at different organizational levels and how they are psychologically working.

Research on HRM and occupational health psychology provides evidence about how organizational resources/practices are related to healthy employees (e.g., vertical and horizontal trust) and healthy outcomes (e.g., organizational and team performance), thus developing healthy and resilient organizations (HEROs). Specifically, Salanova et al. [23] (p. 788) defined HEROs as “those organizations that make systematic, planned, and proactive efforts to improve employees’ and organizational processes and outcomes”. These efforts require the development of healthy organizational practices that result in improvements in resources at the task (autonomy, feedback), social environment (coworker relations, positive leadership), and company (excellent performance) levels. A HERO is an enterprise that balances three components: (1) Healthy organizational resources/practices (e.g., autonomy, feedback, work, and family conciliation); (2) healthy employees (e.g., work engagement, trust); and (3) healthy organizational outcomes (e.g., job performance, commitment, excellent results). In HEROs, organizational practices and team resources are important to make employees feel and perform well, and trust is not only a psychological state because it is also manifested in their consequent behaviors, such as job performance [15,24]. Thus, when organizational practices and team resources are present and are important for employees, they trust their organization (vertical trust) and/or their coworkers (horizontal trust), and consequently, they will try to do their best at the workplace (good performance). The influence of organizational practices and team resources on trust, and then on performance could be explained based on social exchange theory [10]. Practices and resources can build a social exchange mechanism between employees and their organizations (at the organizational level) and teams (at the team level) that influence their levels of trust according to the reciprocity principle, i.e., when organizations or teams give a benefit to the employee, he or she may perceive a sense of “obligation” to reciprocate that benefit then to the organization and/or the team members. When employees perceive high healthy organizational practices, such as work–family balance programs, mobbing prevention, or psychosocial health actions, and/or team resources, such as supportive team climate or autonomy, then they would feel that they are treated well, and consequently, have trust in organizations and/or teams. Moreover, they would also feel the responsibility to reciprocate in terms of better performance at the organizational and team levels. We assume that similar psychological processes take place at the organization (healthy organizational practices lead to vertical trust, and in turn, to higher levels of organizational performance) and team levels (team resources lead to horizontal trust in the team, and in turn, to higher levels of team performance).

Finally, although the relationship between trust and performance is quite well documented at different levels (i.e., individual, team, and organization), there is still a need for research using data from different levels and sources, as well as objective performance measures [25]. Therefore, we used the ROA (objective measure) at the organizational level and performance measures rated by immediate supervisors (different source) at the team level.

### 2.1. Organizational Practices, Vertical Trust and Performance

Organizational practices are defined as “the pattern of planned human resource deployments and activities intended to enable an organization to achieve its goals” [26] (p. 298). These practices are organizational HRM-practices designed to achieve organizational goals and improve psychological and financial health at different organizational levels (i.e., employee, team, and organizational) [23]. As these practices have direct positive effects on psychological wellbeing, we call these “healthy” practices. Research shows that organizations that attempt to implement healthy organizational practices have employees and teams with more positive experiences (e.g., organizational trust [27,28]) and more healthy outputs (e.g., organizational commitment and performance [29,30]). Moreover, healthy organizational practices help the organization to be perceived as a good place to work [31].

Findings based on the European Project EQUAL [23] presents eight relevant HR practices based on corporate social responsibility (CSR) that can be considered. These practices are related to improving work/family balance, skills, and career development, mobbing prevention, improving psychosocial health, open and positive communication, perceived equity, and fomenting corporate social responsibility [23]. Research has shown that increasing vertical trust first requires an investment in healthy organizational practices, and then trust can impact performance [15,32,33]. Several studies show that organizational practices have a relevant effect on psychological wellbeing and trust. For example, in a study conducted among 710 employees nested in 84 groups from 14 small and medium-sized enterprises (SMEs), Salanova et al. [23] showed that organizational practices had a positive impact on employee wellbeing (i.e., engagement, collective efficacy, trust, and resilience), which in turn influenced healthy outcomes (i.e., organizational commitment, job performance). Acosta et al. [11] showed that healthy organizational practices, specifically mobbing prevention, communication, psychosocial health, and work–family balance, can enhance organizational trust at the organizational level. It is important to note that, as Fredrickson and Dutton [32] stated, the positive impact of healthy organizational resources and practices on employees’ health mainly occurs when workers perceive that these organizational strategies are aimed at improving their wellbeing, that is, when employees trust their organization. Moreover, Pirson and Malhotra [34] pointed out that employees with high levels of organizational trust are quite engaged in performing well because they are willing to invest effort and energy in an employer/company that they perceive as competent or benevolent (for example, due to the implementation of healthy practices). Related to this, Xanthopoulou et al. [35] found that healthy employees (i.e., engaged employees) managed to achieve higher objective financial returns for the business. Schneider et al. [30] presented a similar set of results with data aggregated at the organizational level. Over a period of eight years, they found that organizational attitudes (i.e., job satisfaction and satisfaction with job security) predicted financial performance (i.e., ROA). In the systematic review done by Guinot and Chiva [17], they found that a significant number of studies confirmed a positive direct effect of vertical trust on job performance, although non-significant effects were also found for concrete contexts (e.g., non-Western cultures), specific measures of job performance (e.g., extra-role performance), and some types of subordinates and leaders. This systematic review concluded that there is a lack of empirical analysis of the mediating role that vertical trust plays in the team’s and organizational performance, and that future studies should continue to analyze the differences in this direct relationship according to the leadership referent of trust (i.e., trust in top leaders, like in the current study).

Interestingly, when unhealthy practices are implemented by organizations, the opposite occurs. For example, Wells and Kipnis [36] found that in 267 subordinates, distrust in their managers was related to the use of strong methods of influence, less interaction between colleagues, and fewer attempts to influence each other. All these bad practices predicted subordinates’ lack of organizational trust in their managers. Furthermore, the study developed by Saunders et al. [37] showed that when workers are distrustful, there are several organizational interventions available to build trust that might also be necessary to reduce distrust. The most relevant ones focus on positive and congruent communication between leaders and employees and look for consistency in the managers’ actions, ensuring that promises are kept, and that interventions and subsequent actions are truthfully communicated and properly reported (i.e., congruence, integrity, consistency).

**Hypothesis** **1.** *At the organizational level, vertical trust is mediating the relationship between healthy organizational practices and organizational performance.*

### 2.2. Team Resources, Horizontal Trust and Performance

Regarding team resources, Lyubomirsky et al. [38] proposed that resources help people to thrive and succeed at work, causing them to be “healthier” in their social relationships and personal wellbeing. Moreover, previous research indicated that social resources are specific types of resources that may act as antecedents of wellbeing (I.e., trust). These social resources are related to the interaction and interdependence among the members of teams. For instance, Hakanen et al. [39] found that teachers who showed better social team resources (i.e., innovation climate, support from leaders and colleagues) experienced higher degrees of psychological wellbeing than teachers with fewer social resources. Similar results were found by Martínez-Tur et al. [15], indicating that reciprocity of trust between managers and teams improves wellbeing and quality of service as an indicator of job performance. In another study, Harms et al. [40] showed that the relationships between the followers’ (anxious or avoidant) attachment orientation and workplace outcomes (i.e., job stress and citizenship behaviors) were mediated by trust in their supervisors.

Horizontal trust refers to the aggregate levels of trust that team members have in their fellow teammates [41], and it focuses on the team level. Considering that organizations have become flatter, and more team-focused, teams play an important role in increasing performance [42] and psychosocial health [43,44]. When organizations facilitate positive collaborative working practices, team performance improves [45]. Team performance is defined as the extent to which a team accomplishes its goal or mission [46] being in-role or extra-role performance [47] or task and contextual performance, respectively. In this regard, task performance comprises activities related to the formal job and task description, and contextual performance consists of activities that exceed the prescribed tasks employees must do (e.g., voluntary overtime, help colleagues). These two complementary job performance types simultaneously provide a comprehensive view of team performance. In the team dynamics literature, trust among coworkers (i.e., horizontal trust) is seen as a critical mechanism in explaining how team resources are related to successful team performance [24]. Horizontal trust leads employees to behave based on their faith in the words and actions of their peers [48]. This means that, if people trust others on their team, they seek interaction with them, and they are more likely “to like what they like and see what they see”, thus sharing definitions of importance and furthering integration between them [49]. Furthermore, horizontal trust is related to other important outcomes, such as turnover intention [50] and organizational commitment [51,52]. In a meta-analysis with a sample of 112 independent studies (N = 7763 teams), De Jong et al. [53] showed that horizontal trust is positively related to team performance and has an above-average impact (*p* = 0.30). Specifically, they showed very convincingly that the magnitude of the effect size estimated for the relationship of horizontal trust and team performance is the highest compared to other team level constructs, and this relationship is stronger when the trust referent is the team (“I trust in my team”) instead of an individual teammate (“I trust teammate X”). They conclude that these results have important implications for how trust is working in teams, and more future research is needed on this topic, as well as how team trust perceptions are formed in teams. In our study, we assume that horizontal trust is positively related to team performance and that team resources are the drivers of this trust. When team members feel that they are benefitted from these resources (i.e., supportive social climate), they trust their team and try to reciprocate by investing energy in the team and getting a better team performance, in line with social exchange theory.

**Hypothesis** **2.** *At the team level, horizontal trust is mediating the relationship between team resources and team performance.*

### 2.3. Cross-Level Effects on Trust and Performance

Finally, although there is not much research on trust as a psychological mechanism that explains cross-levels effects between antecedents and consequences of trust [17], based on social exchange theory, we could expect that higher levels of healthy organizational practices positively influence employees’ levels of trust in their top management (vertical trust) which in turn enhance team performance. In addition, these practices could also influence horizontal trust, and in turn, enhance team performance too (as a way of showing reciprocity). We formulated the following cross-level hypotheses:

**Hypothesis** **3.** *Healthy organizational practices (organizational level) are positively related to team performance (team level) above and beyond horizontal trust (team level).*

**Hypothesis** **4.** *Healthy organizational practices (organizational level) are positively related to horizontal trust (team level) above and beyond team resources (team level).*

**Hypothesis** **5.** *Vertical trust (organizational level) is positively related to team performance (team level) above and beyond horizontal trust (team level).*

**Figure 1:**
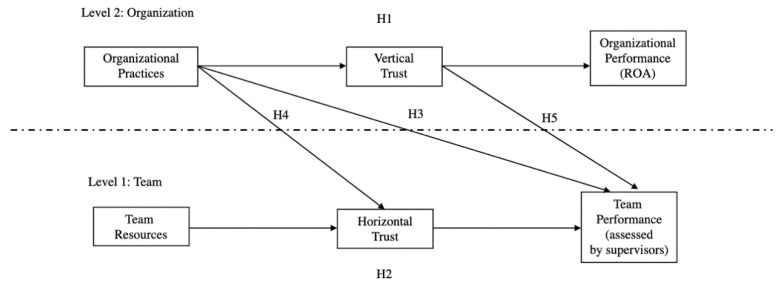
Research model and study hypotheses.

In Figure 1, we draw the research model with variables of the study at different levels of analysis, as well as the study hypotheses.

## 3. Materials and Methods

### 3.1. Data Collection

The study sample consisted of 890 employees (average response rate per organization was 62%) nested within 177 teams and their 177 immediate supervisors from 31 Spanish companies. Of the employees, 58% were women, and 79% had a tenured contract. Their average tenure in the company was six years (SD = 4.05). Of the supervisors, 51% were female, and 86% had a tenured contract. Their average tenure in the company was 15 years (SD = 12.21). The average number of people in a team was five (SD = 2.35), and organizations had 48 employees on average (SD = 32.44). Organizations also differed in terms of economic sector—86% operated in the service sector and 14% in industry.

The Human Resource Managers or CEOs of the participating organizations provided their employees and team supervisors with information about the project through different means (e.g., company bulletin board, meetings, intranet). In addition, researchers further explained the project by means of information meetings. Employees and supervisors completed a self-report questionnaire with questions about their organizations and teams, focusing on their organizational and team perceptions. The researchers distributed the questionnaire themselves, and it took approximately 30 min to fill it out. In order to guarantee that workers were familiar with the functioning of the organization, only workers with an organizational tenure of at least six months were included in the study because it takes at least a couple of months for new employees to get settled into their organization. Confidentiality of the responses was guaranteed. In this way, the research team ensured strict compliance with applicable regulations, especially about the utmost confidentiality in handling data.

### 3.2. Measures 

#### 3.2.1. Healthy Organizational Practices

These practices were assessed at the organizational level with five items that represent four practices included in the HERO (healthy and resilient organizations) questionnaire [23]. Although eight healthy organizational practices were included in the original survey, a previous study conducted by Acosta et al. [11] demonstrated that four of them are positively related to trust: Work–family balance (one item; ‘In the last year, practices and strategies have been introduced in this organization to facilitate the work–family balance and the private lives of its employees’); mobbing prevention (one item; ‘In the last year, practices and strategies have been introduced in this organization to prevent mobbing at work’); psychosocial health (one item; ‘In the last year, practices and strategies have been introduced in this organization to ensure wellbeing and quality of life at work’); and organizational communication (two items; ‘In the last year, practices and strategies have been introduced in this organization to facilitate communication from management to workers’; ‘In the last year, practices and strategies have been introduced in this organization to ensure that information about the organizational goals is given to everyone who needs to know about them’). Internal consistency for the scale was 0.84, which is above the cut-off point of 0.70 [54]. We used a 7point Likert-type scale ranging from 0 (never) to 6 (always). In order to draw respondents’ attention from the individual level to the organizational level, all the items were focused on organizational perceptions.

#### 3.2.2. Vertical Trust

It was assessed at the organizational level with four items based on Huff and Kelley [55]. An example of an item is: ‘In this organization, subordinates have a great deal of trust in their supervisors and top managers’. Internal consistency was 0.90, which is above the cut-off point of 0.70 [54]. Respondents answered using a 7-point Likert-type scale ranging from 0 (strongly disagree) to 6 (strongly agree). Once again, in order to draw respondents’ attention from the individual level to the organizational level, all the items focused on organizational perceptions.

#### 3.2.3. ROA

It was obtained at the organizational level from the SABI database (http://sabi.bvdep.com) (accessed on 20 November 2011). This objective database contains general and financial information from each organization. ROA is an independent variable indicating how profitable a company is regarding its total assets, and it refers to how efficient management is when using its assets to generate earnings. ROA is calculated when dividing a company’s annual earnings by its total assets, obtaining a percentage. We focused on ROA as a financial indicator that is more stable and consistent over time [30]. Thus, return on assets measures a company’s earnings in relation to all the resources it has at its disposal.

#### 3.2.4. Team Resources

Team resources were assessed at the team level with 12 items belonging to four different scales included in the HERO questionnaire [23]. These are: Autonomy (three items—e.g., ‘In my team, we decide when to begin, finish, and the order in which we do the tasks’; alpha = 0.70); coordination (three items—e.g., ‘In my team, we coordinate our activities’; alpha = 0.77); feedback (three items—e.g., ‘In my team, the work we do gives us a lot of information about how well you are doing’; alpha = 0.69); and supportive team climate (three items—e.g., ‘In my team, constructive criticism is rewarded’; alpha = 0.77). Respondents answered using a 7-point Likert-type scale ranging from 0 (never) to 6 (always).

#### 3.2.5. Horizontal Trust

It was assessed at the team level with four items based on McAllister [56]. An example item is: ‘In my team, we can share our ideas, emotions, and hopes’. Internal consistency was 0.85, which is above the cut-off point of 0.70 [54]. A 7-point Likert-type scale was used by respondents, ranging from 0 (strongly disagree) to 6 (strongly agree). Here, in order to draw respondents’ attention from the individual level to the team level, all the items focused on team perceptions.

#### 3.2.6. Team Performance (Rated by Immediate Supervisor)

It was assessed at the team level by immediate supervisors using a six-item scale adapted from Goodman and Svyantek [47]. We considered two different scales: In-role performance (three items—e.g., ‘The team that I supervise achieves its work goals’; alpha = 0.84) and extra-role performance (three items—e.g., ‘In the team that I supervise, employees help each other when somebody is overloaded’; alpha = 0.71). Team supervisors answered using a 7-point Likert-type scale ranging from 0 (strongly disagree) to 6 (strongly agree).

#### 3.2.7. Control Variables

Teamwork was assessed with three items (e.g., ‘My team has well-defined teamwork goals’; alpha = 0.75) [23]. We use teamwork as a control variable to guarantee that each team shares a common goal with interrelated tasks. Furthermore, we included team size (i.e., the total number of members per team) at the team level of analysis because previous studies have consistently shown that it affects group dynamics and performance (i.e., cohesion; team goals) [57,58]. Organizational size (i.e., the total number of employees per organization) was also included as a control variable at the organizational level of analysis because, in this study, we are considering enterprises of different sizes.

More information about the scales and sources are in Appendix A.

### 3.3. Analytical Strategy

In this study, the questionnaire measures three team level variables and two organizational level variables from two different sources of information. Using the organization as a referent, the employees assessed healthy organizational practices and vertical trust. Team resources and horizontal trust were assessed by the employees using their team as a referent. Team performance was assessed by team supervisors using the “team” as a referent.

Because the variables in our research model—except for ROA—were aggregates of lower-level shared perceptions, interrater reliability and interrater agreement indices had to be computed [59]. Employees’ agreement was assessed using a two-fold approach: (1) ICC_1_ was calculated following a consistency-based method. Although there is no fixed cut-off point for ICC_1_, a value of 0.01 might be considered a small effect, a value of 0.10 might be considered a medium effect, and values above 0.25 might be considered a large effect [60]; (2) following a consensus-based approach, we computed the Average Deviation Index (AD_M(J)_; [61], where agreement among team members or the organization is established when AD_M(J)_ is equal to or less than 1 for 7-point Likert-type scales [61]. We also computed different Analyses of Variance (ANOVA) to find out whether there was significant between-group discrimination on the measures at the organizational and team levels. All the variables showed between small and medium effects for ICC_1_, and ANOVA analyses indicated significant discrimination of variables between groups or organizations (from 0.18 to 0.47) (see Table 1). AD_M(J)_ indices showed values of less than 1 (average AD_M(J)_ was 0.80). Overall aggregation results indicated agreement at the organizational level regarding employees’ perceptions of healthy organizational practices and vertical trust. In a similar way, aggregation indices also showed an adequate level of agreement on the team level variables, i.e., healthy team resources, horizontal trust, and teamwork. Finally, we computed descriptive statistics and intercorrelations among the scales based on data aggregated at the team level and the organizational level, respectively.

### 3.4. Data Analyses

Harman’s single factor test [62] was performed for the employee variables in the study to test for bias, due to common method variance. However, it is important to keep in mind that the dependent variables in our database (i.e., ROA and supervisor perceptions of performance) and the independent variables came from different sources. Finally, we used regression analyses by PASW 18.0 as a previous test of Hypotheses 1 (at the organizational level) and 2 (at the team level). To test the mediation process of Hypothesis 1, the bootstrapping procedure was used [63]. This method is recommended to examine mediation in small sample sizes [64], and it offers an empirical means of determining the significance of statistical estimates [65]. We used the bootstrapping procedure in AMOS 18.0 (Analyses of Moment Structures; [66].

To test Hypothesis 2, SEM by AMOS 18.0 [66] was used. Team resources (i.e., autonomy, coordination, feedback, and supportive team climate) comprised one indicator. Horizontal trust (i.e., four items) comprised another indicator. Finally, performance (supervisor-rated performance) comprised the final indicator. For all these variables, the error variance of each indicator was constrained in all the models to avoid unidentified problems by using the formula, (1−α) × σ. We used maximum likelihood estimation methods. Two absolute goodness-of-fit indices were assessed to evaluate the goodness-of-fit of the models: (1) The χ2 goodness-of-fit statistic and (2) the Root Mean Square Error of Approximation (RMSEA). The χ2 goodness-of-fit index is sensitive to sample size; for this reason, the use of relative goodness-of-fit measures is recommended [67]. Thus, four relative goodness-of-fit indices were used: (1) Comparative Fit Index (CFI), (2) Normed Fit Index (NFI); (3) Tucker-Lewis Index (TLI, also called the Non-Normed Fit Index); and (4) Incremental Fit Index (IFI). For RMSEA, values smaller than 0.05 indicated an excellent fit, 0.08 indicated an acceptable fit, and values greater than 0.10 led to model rejection [68]. Regarding the relative fit indices, values greater than 0.90 indicated a good fit [69]. The mediation effect was assessed using the approach developed by Baron and Kenny [70] and the Sobel test [71].

In the current study, Hypotheses 3–5 were tested by means of random coefficient modeling or hierarchical linear modeling [72]. The Intraclass Correlation Coefficient (ICC) is a measure of non-independence, testing the percentage of variance explained by a set of contextual variables [73]. The higher the ICC, the greater the amount of variability in the dependent variable that can be explained by variables from the higher level of analysis (i.e., the organization in the current study). A baseline ANOVA model was computed to evaluate non-independence ICC as a procedure for comparing models, and to evaluate the variance percentages for the levels involved in the analyses [74].

Apart from the baseline ANOVA model, two other models were tested, following a step-by-step approach using maximum likelihood, as implemented by LISREL 8.8 [75]. (1) We conducted a random-coefficient regression model (Model 1) in which random coefficients were freed to vary between organizations. Group-level controls and predictors were also included in the model equation. This model provides tests of lower-level predictors, while considering the nested structure of the data and controlling for lower-level covariates. (2) The intercepts-as-outcomes model (Model 2) included organizational level controls and predictors in the equation for the intercept. For the random-coefficient regression model, team level variables were grand-mean centered [76].

## 4. Results

### 4.1. Correlation and Confirmatory Factor Analyses

Means, standard deviations, Cronbach’s alphas, and intercorrelations among the variables at the individual, team, and organizational levels are displayed in Table 1, Table 2 and Table 3, respectively. As expected, all study variables were positively and significantly correlated. The results of Harman’s single factor test [62] of the individual database (N = 871) revealed a poor fit to the data, χ^2^(18) = 169.658, *p* = 0.000, RMSEA = 0.201, CFI = 0.676, NFI = 0.587, TLI = 0.565, IFI = 0.678. To avoid the problems related to the use of Harman’s single factor test [62], we compared the results of the one latent factor model with a model considering four latent factors. Results showed a significantly lower fit of the model with one single factor compared to the model with multiple latent factors, Delta χ^2^(2) = 109.424, *p* < 0.001. Common method variance is not a serious problem in this study.

### 4.2. Hypothesis Testing

Hypotheses 1 and 2, were confirmed through regression analysis and SEM. The positive relationship between healthy organizational practices and vertical trust at the organizational level of analysis, was confirmed (β = 0.81, *p* < 0.001). Moreover, organizational size was negatively and significantly related to vertical trust (β = –0.04, *p* < 0.001). Healthy organizational practices explained 71% of the variance in vertical trust (see Table 4). Moreover, vertical trust is positively related to organizational performance (financial indicator, return on assets; ROA) (β = 0.47, *p* < 0.001). Organizational size was not significantly related to organizational performance (β = 0.03, ns), and vertical trust explained 11% of the variance in ROA (see Table 4). Moreover, our results showed that team resources are positively related to horizontal trust at the team level of analysis (β = 0.62, *p* < 0.001), whereas team size was not significantly related to horizontal trust (β = −0.01, ns). Team resources explained 34% of the variance in horizontal trust. Moreover, horizontal trust is positively related to (supervisor-rated) team performance (β = 0.40, *p* < 0.001). Again, team size was not significantly related to team performance (β = 0.02, ns). Horizontal trust explained 26% of the variance in team performance (see Table 5).

To test Hypothesis 1, in which vertical trust mediates the relationship between healthy organizational practices and ROA at the organizational level, we used a bootstrapping procedure, also controlling for organizational size. The procedure involves repeated random sampling observations with replacement from the data and calculation of the statistic of interest in each resample. In our case, we considered a resample of N = 500. Results indicated that vertical trust fully mediated the relationship between healthy organizational practices and ROA. The non-significant direct relationship between healthy organizational practices and ROA indicated that there was a full mediation. The 95% confidence interval of the mediation model does not include 0, which indicates that the proposed model is statistically significant [63] (see Table 6). To confirm the mediation effects, we performed the Sobel Test [71], which showed a significant result (Sobel t = 2.52, *p* = 0.001) (see Table 6).

To test Hypothesis 2, in which horizontal trust mediates the relationship between team resources and performance (supervisor-rated performance) at the team level, we performed SEM-analyses with AMOS. Two models were tested, (M1): Full mediation, and (M2): Partial mediation. Teamwork and team size were included as control variables. Table 7 shows the results of the SEM analyses conducted to test the relationships among healthy team practices, horizontal trust, and team performance. The findings of these analyses indicate that M1 and M2 fitted the data well. M1: χ^2^(11) = 15.52, RMSEA = 0.04, CFI = 0.97, NFI = 0.91, TLI = 0.89, IFI = 0.92. M2: χ^2^(10) = 12.04, RMSEA = 0.03, CFI = 0.98, NFI = 0.91, TLI = 0.89, IFI = 0.90. The difference between the two models was not significant, Delta χ^2^(1) = 3.48, ns, which means that both models fit the data well. These results support M1 because it is more parsimonious than M2. To confirm the mediation effect, we performed the Sobel Test [71], which yielded a significant result (Sobel t = 3.55, *p* < 0.001). These results provide evidence for M1; that is, horizontal trust fully mediates the relationship between team resources and supervisor rated team performance. As expected, team resources have a positive and significant relationship with horizontal trust (β = 0.62, *p* < 0.001), which in turn is positively and significantly related to supervisor-rated team performance (β = 0.40, *p* < 0.001). It is interesting to note that team resources explain 34% of the variance in horizontal trust (R^2^ = 0.34), which in turn explains 26% of the variance in team performance (R^2^ = 0.26).

According to Hypothesis 3, healthy organizational practices are positively related to team performance above and beyond horizontal trust. Table 8 includes the results for the hierarchical linear models predicting performance. Model 1 included horizontal trust in the equation, along with the team level control variables (i.e., teamwork, team size). Results for Model 1 show that horizontal trust has a positive and significant relationship with team performance (β = 0.31, *p* = 0.05). Model 2 included organizational level variables to test for cross-level effects, that is, healthy organizational practices and organizational size as control variables. Unexpectedly, healthy organizational practices were not significantly related to team performance (β = −0.03, ns). Therefore, Hypothesis 3 was not confirmed.

According to Hypothesis 4, healthy organizational practices are positively related to horizontal trust above and beyond team resources. Model 1 included team resources in the equation, along with the team level control variables (i.e., teamwork, team size). Model 2 included organizational level variables to test for cross-level effects, that is, healthy organizational practices and organizational size as control variables. Nevertheless, it turned out that the baseline ANOVA model was 3%. This means that only 3% of the variance in horizontal trust is explained by variables at other levels. In our case, 3% of the variance is explained by variables at the organizational level. According to Bliese [73], more than 5% is needed to allow hierarchical linear modeling to be conducted. Therefore, this cross-level effect was not tested because one preliminary condition, that is, the ANOVA model, was not favorable. Hypothesis 4, therefore, was not confirmed.

According to Hypothesis 5, vertical trust is positively related to team performance above and beyond horizontal trust. Table 9 includes results for the hierarchical linear models predicting team performance. Model 1 included horizontal trust in the equation, along with the team level control variables (i.e., teamwork, team size). Model 1 results again show that horizontal trust has a positive and significant relationship with team performance (β = 0.31, *p* = 0.05). Model 2 included organizational level variables to test for cross-level effects, that is, vertical trust and organizational size as control variables. Unexpectedly, again vertical trust was not significantly related to team performance (β = 0.03, ns). Thus, Hypothesis 5 was not confirmed.

**Figure 2:**
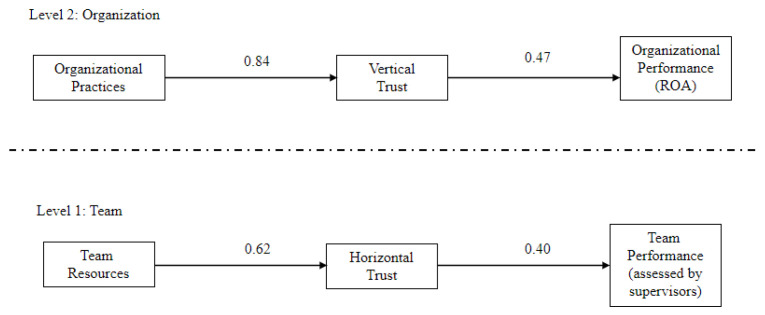
Final Model: Multilevel analyses (N = 890 employees nested in N level 2—31 companies; N level 1—177 teams with employees and their immediate supervisors).

Hence, from these results, it can be concluded that there are no cross-level effects of organization level variables on the team level outcomes. That is, there are two different, parallel processes where different types of trust (vertical and horizontal) have a mediating role. At the team level, horizontal trust plays a fully mediating role between team resources and team performance. At the organizational level, vertical trust plays a fully mediating role between healthy organizational practices and ROA (see Figure 2).

## 5. Discussion

This study contributes to our understanding of the role of trust as a psychological mechanism that explains how healthy organizational practices and team resources are related to performance at different levels of analysis (organization and team levels, respectively), following a multilevel-multireferent framework.

### 5.1. Theoretical Implications

The current study offers evidence of: (a) At the organizational level, the fully mediating role of vertical trust in the relationship between healthy organizational practices and financial performance (ROA) (Hypothesis 1); and (b) at the team level, the fully mediating role of horizontal trust in the relationship between team resources and (supervisor-rated) team performance (Hypothesis 2). Contrary to our expectations, we did not find evidence for cross-level effects (Hypotheses 3–5).

Through regression analysis, with data aggregated at the organizational level, we confirmed first the relationship between healthy organizational practices implemented through HRM (i.e., work–family balance, mobbing prevention, psychosocial health, and communication) and vertical trust. This result is consistent with previous studies that pointed out that when organizations develop practices oriented toward improving the wellbeing of their employees, trust emerges [11,17,26,77]. Employees’ trust in their top managers acts as an explanatory factor, which translates the benefits of certain organizational practices into better organizational performance. Inversely, also, this organizational trust could be diminished when employees feel not been reciprocated, such as feeling insecurity towards their jobs [78] being trust also a mediator psychological mechanism to explain (in this case) how job insecurity is related to performance.

Our results provide important information for HR practitioners about how to develop trust in their organizations, for example, by means of work–family balance practices (e.g., teleworking) that allow employees/teams to manage their personal lives and their careers.

Following the reciprocity effect based on social exchange theory, when employees feel beneficed by practices that their organization implements to increase their psychological health, their levels of vertical trust increase, and they feel the obligation to reciprocate to the organization via enhancing organizational performance. This result is consistent with the study by Schneider et al. [30], which shows that employee attitudes at work are related to the financial performance of organizations. In our case, if employees trust their organizations, financial performance is improved. Even more, our analysis revealed that organizational trust fully mediated the relationship between healthy organizational practices implemented through HRM (i.e., work/family interaction, mobbing prevention, psychological wellbeing, and positive communication) and healthy outcomes (i.e., organizational financial performance). These results extend previous research conducted at the individual level of analysis, where healthy organizational practices are positively related to healthy employees and healthy organizational outcomes [12, 14, 38). The present study used aggregated perceptions at the organizational level, as proposed by Fulmer and Gelfand [3], and external (objective) criteria for performance, that is, ROA. This result, therefore, confirms the key role of vertical trust in organizational processes for competitive advantage [79]. Thus, vertical trust is a pivotal element for employees to feel good and perform well at work. We can conclude that organizations must foster trust between employees and top managers because this will have a positive impact on organizational performance (i.e., financial performance).

Hypothesis 2 was tested first through regression analysis with data aggregated at the team level. We confirmed the relationship between team resources (autonomy, coordination, feedback, and supportive team climate) and horizontal trust. This result shows that when teams share positive beliefs about their team resources, such as coordination or a socially supporting climate, this allows trust in coworkers to emerge. This result is in line with Torrente et al. [80], who pointed out that when teams perceive that they have team resources, healthy employee perceptions emerge (i.e., teamwork engagement). Secondly, through regression analysis with data aggregated at the team level, we confirmed the positive relationship between horizontal trust and (supervisor-rated) team performance. Following Whitman et al.’s [43] recommendation to focus on a collective level of analysis, the present study used ratings of team performance provided by the supervisor. When team members trust their coworkers, supervisory perceptions of team performance are more favorable. This result also confirms previous studies by Costa [24], who pointed out that high work-team trust leads to high team task performance. Finally, results of the SEM analyses to test the mediation effect revealed that horizontal trust fully mediated the relationship between team resources (i.e., autonomy, coordination, feedback, and supportive team climate) and performance tested at the team level, pointing out the key role of trust at the team level too. In other words, when teams perceive that they have autonomy, are coordinated, receive feedback, and work in a supportive climate, horizontal trust emerges among coworkers, and according to the social exchange theory, employees feel that they must reciprocate and increase efforts to have a better team performance (as assessed in our case by their immediate supervisor). Thus, organizations must also consider implementing job resources in their teams to develop horizontal trust because if the members of a team trust each other, team performance will be better.

Hypotheses 3–5 were not supported. These hypotheses considered the cross-level effects between the variables included in this study. Previous studies have demonstrated the positive relationship between organizational practices and performance (28, 38) or between healthy employees and performance [39,80], considering individual or team perceptions of employees’ performance. However, using a multilevel framework, in the present study, these relationships were not found. Our results show two parallel motivational processes, where trust plays a key role as a mediator at the team (i.e., horizontal trust) and organizational (i.e., vertical trust) levels. Therefore, organizations must implement both healthy organizational practices (work–family balance; mobbing prevention, psychosocial health, and communication) and team job resources (i.e., autonomy, coordination, feedback, and supportive team climate) at the same time, in order to develop trust at different organizational levels (i.e., vertical trust and horizontal trust), and thus, obtain high levels of performance (i.e., organizational financial performance and supervisor-rated team performance).

In sum, the present study contributes theoretically to previous organizational trust research in two ways. First, it extends the body of knowledge about the key role of organizational trust (i.e., horizontal trust and vertical trust) in the relationship between healthy organizational practices and team resources and performance, using data aggregated at the organizational and team levels. The positive relationships found support and extend social exchange theory [19]. Previous research based on trust as a product of a social exchange process [51,52] found positive relationships between organizational trust dimensions and positive outcomes, such as organizational commitment or identification. In our study, at the organizational level, employees generate “(vertical) trust” in the organization when they receive healthy practices, and “in exchange”, they perform better for the benefit of the company. In addition, employees generate “(horizontal) trust” when they receive positive resources from the team, such as autonomy, positive feedback, and supportive team positive climate. In turn, they perform better as a team as a type of benefit “exchange”.

Second, although it has been recognized that trust in organizations occurs at multiple levels [19] and using different referents [3], there are no clear empirical findings on how trust in organizations operates simultaneously at different levels and with different referents [22]. The main finding of the current research is that when studying organizational trust simultaneously at the team and organization levels, the same social exchange process occurs at both levels. However, these processes occur in parallel because we did not find cross-level effects of trust. This result points to “a positive mirror effect”, where organizational and team social exchange processes of trust are operating in the same way, but in parallel (as a mirror). This finding agrees with the assumption of construct quasi-isomorphism pointed out by Fulmer and Gelfand [3]. Our results provide evidence for theoretical quasi-isomorphism based on social exchange theory, as well as functional quasi-isomorphism, because our relationships are similar at different levels.

### 5.2. Practical Implications

From a practical point of view, our findings provide contributions for practitioners in HRM and business strategy, as well as managers in organizations, with a more holistic understanding of the links between healthy practices and resources, organizational trust, and performance. Our results support the effectiveness of different healthy practices and actions that could be carried out through HRM to build organizational trust in teams and the organization from a perspective based on continuous promotion actions. The organizational level results show the relevance of investing in work/family balance, mobbing prevention, psychological wellbeing, and positive organizational communication, because doing that, then employees will interpret investment in these practices as a sign that the organization is concerned about their wellbeing, and consequently, (vertically) trust in the organization will be enhanced, resulting in the improved financial performance of the organization (i.e., ROA). The team level results show a similar tendency: The relevance of investing in autonomy, coordination, feedback, and a supportive team climate, enhances (horizontal) trust and them an improved supervisor-rated team performance.

### 5.3. Limitations and Suggestions for Future Research

The present study has some limitations. The first one is that some of the data were obtained through self-reports. However, aggregate rather than individual perceptions of teams and organizations have been considered, and a multilevel framework was used, as proposed by Hox [74]. Moreover, two external and objective criteria were considered (i.e., ROA and supervisor-rated team performance) to minimize the common method variance bias, as recently recommended by Hox [74].

Second, the employee data in this study are mainly cross-sectional. However, we included the ROA indicator for the following year as a dependent variable at the organizational level of analysis. Future studies should test the model by including data collected in different waves, to truly test the relationship between healthy organizational resources and practices, organizational trust (i.e., vertical trust and horizontal trust), and healthy organizational outcomes over time.

Thirdly we agree with Fulmer and Gelfand [3] that another interesting topic for future research would be the trust climate construct, using direct consensus or referent-shift models. Using longitudinal designs, we could increase the knowledge about multilevel antecedents and consequences of trust climate, as well as the influence of the strength of the trust climate on important business outcomes, such as performance.

Finally, we also could consider other more objective organizational performance indicators, such as MTB, to validate the results obtained by ROA.

## 6. Conclusions

In conclusion, our findings suggest that healthy organizational practices and team resources influence organizational/team performance via organizational trust (vertical vs. horizontal). Two motivational and parallel processes were found. First, at the organizational level, vertical trust plays a fully mediating role between healthy organizational practices and organizational performance (ROA). Second, at the team level, horizontal trust plays a fully mediating role between healthy team practices and (supervisor-rated) team performance. Researchers, corporate managers, stakeholders, and practitioners should use these results to implement and design organizational practices and team resources that generate a sense of trust among employees and then contribute to the emergence of ‘creating’ positive employees and business outcomes. The implications of the research for sample firms goes around the idea to invest in healthy organizational and team resources to be more competitive and to have more healthy employees and better performance. They can benefit from the outcomes of this research by incorporating the evaluation, assessment, and improvement of the three elements that characterize a HERO: Healthy organizational resources and practices, healthy employees by means of vertical and horizontal trust, and better organizational results, such as performance at the organization and team levels. Firms in a different macro-economic setting can learn from this research. Concretely the key lesson is that being competitive depends on the efforts that the organizations make to create a positive, psychologically trusting company that promotes a healthy and competitive workforce.

## Figures and Tables

**Table 1:** Means, standard deviations, aggregation indices, and intercorrelations among the study variables at the individual level (N = 890).

Variables	M	SD	α	ICC1	ADM(J)	1	2	3	4	5	6	7	8
1. Healthy Org. Practices	3.44	1.44	0.84	0.45	0.89	-							
2. Vertical Trust	3.88	1.34	0.90	0.52	0.65	0.68 ***	-						
3. ROA	1.60	19.77	-	-	-	0.14 ***	0.19 ***	-					
4. Team Resources	4.33	1.00	0.77	0.32	0.78	0.49 ***	0.44 ***	0.05	-				
5. Horizontal Trust	4.27	1.13	0.85	0.28	0.64	0.47 ***	0.57 ***	0.10 **	0.51 ***	-			
6. Team Performance	4.94	0.86	0.71	0.18	0.91	0.30 ***	0.28 ***	0.14 ***	0.46 ***	0.45 ***	-		
7. Teamwork	4.83	1.16	0.75	0.47	0.95	0.4 ***	0.41 ***	0.06	0.70 ***	0.45 ***	0.45 ***	-	
8. Organizational size	48.37	31.67	-	-	-	−0.80 *	-0.23 ***	0.00	−0.00	−0.61	0.13 ***	−0.05	-
9. Team size	5.35	2.42	-	-	-	−0.32	−0.12 ***	0.13 ***	0.00	−0.58	0.12 ***	−0.01	0.038 ***

*Notes.* M = mean, SD = Standard Deviation, α = Cronbach’s alpha, ICC1 = Intraclass Correlation Coefficient, ADM(J) = Average Deviation Index, * *p* < 0.05, ** *p* < 0.01, *** *p* < 0.001.

**Table 2:** Means, standard deviations, and intercorrelations among the study variables at the team level (N = 177).

Variables	M	SD	1	2	3	4
1. Team Resources	4.38	0.69	-			
2. Horizontal Trust	4.33	0.74	0.58 ***	-		
3. Team Performance	4.91	0.58	0.50 ***	0.50 ***	-	
4. Teamwork	4.88	0.81	0.81 ***	0.54 ***	0.49 ***	-
5. Team size	5.03	4.2	−0.08	−0.12	0.006	−0.90

*Notes.* M = mean, SD = Standard Deviation, *** *p* < 0.001.

**Table 3:** Means, standard deviations, and intercorrelations among the study variables at the organizational level (N = 31).

Variables	M	SD	1	2	3
1. Healthy Org. Practices	3.45	0.56	-		
2. Vertical Trust	3.94	0.60	0.81 ***	-	
3. ROA	0.71	17.22	0.25 ***	0.32 **	-
4. Organizational size	0.48	34.50	−0.18 *	−0.39 ***	−0.02

*Notes.* M = mean, SD = Standard Deviation, * *p* < 0.05, ** *p* < 0.01, *** *p* < 0.001.

**Table 4:** Regression analyses by aggregating data (N = 31).

		Vertical Trust	
Predictor Variables	B	SE B	β
1. Healthy Org. Practices	0.81	0.04	0.76 ***
2. Organizational Size	−0.04	0.00	−0.26 ***
R^2^ = 0.71 ∆R^2^ = 0.71			
		ROA 2010	
Predictor variables	B	SE B	β
1. Vertical Trust	0.47	0.05	0.33 ***
2. Organizational Size	0.03	0.05	0.05
R^2^ = 0.11 ∆R^2^ = 0.10			

*Notes.* B = Beta, SE B = Standard Error of Beta, β = Adjusted Beta, R^2^ = Coefficient of determination, ∆R^2^ = Adjusted Coefficient of Determination, *** *p* < 0.001.

**Table 5:** Regression analyses by aggregating data (N = 177).

		Horizontal Trust	
Predictor Variables	B	SE B	β
1. Team Resources	0.62	0.07	0.57 ***
2. Team Size	−0.01	0.01	−0.07
R^2^ = 0.34 ∆R^2^ = 0.33			
		Performance (supervisor-rated)	
Predictor variables	B	SE B	β
1. Horizontal Trust	0.40	0.05	0.52 ***
2. Team Size	0.02	0.00	0.12
R^2^ = 0.26 ∆R^2^ = 0.26			

*Notes.* B = Beta, SE B = Standard Error of Beta, β = Adjusted Beta, R^2^ = Coefficient of determination, ∆R^2^ = Adjusted Coefficient of Determination, *** *p* < 0.001.

**Table 6:** Bootstrapping for healthy organizational practices, vertical trust, and ROA-2010 mediation model aggregated data (N = 31 companies).

	Bootstrap		BC 95% CI		
Indirect Effects	Estimate	SE	CI Lower	CI Upper	*p*
***ROA-2010***					
Healthy Org. Practices	1.17	0.30	0.81	1.69	0.01

*Notes.* Number of bootstrap resamples = 500, BC 95% CI = Confidence Interval, SE = Standard Error, CI Lower = Confidence Interval Lower, CI Upper = Confidence Interval Upper, *p* = Significance.

**Table 7:** Fit indices for structural equation models by aggregated data at the team level of analysis (N = 177 teams).

Models	χ^2^	*gl*	RMSEA	CFI	NFI	TLI	IFI	∆χ^2^	*gl*	∆RMSEA	∆CFI	∆NFI	∆TLI	∆IFI
M1	15.52	11	0.04	0.97	0.91	0.89	0.92							
M2	12.04	10	0.03	0.98	0.91	0.89	0.90							
Diff. M2-M1								3.48	1	0.01	0.01	0.00	0.00	0.02

*Notes.* M1 = Model 1, M2 = Model 2, 2 = Chi-square, df = degree of freedom, RMSEA = Root Mean Square Error of Approximation, CFI = Comparative Fit Index, NFI = Normed Fit Index, TLI = Tucker-Lewis Index, IFI = Incremental Fit Index, Diff. and ∆ =Differences.

**Table 8:** Results for the hierarchical linear models predicting performance (ICC = 12%).

Parameters	Model 1	Model 2
Intercept	4.90 *** (0.05)	4.91 *** (0.05)
Level 1 (teams)		
Teamwork	0.19 *** (0.04)	0.19 *** (0.05)
Team size	−0.01 (0.00)	−0.01 (0.00)
Horizontal Trust	0.31 *** (0.05)	0.31 *** (0.05)
Level 2 (organizations)		
Organizational size		−0.00 (0.00)
Healthy Org. Practices		−0.03 (0.11)

*Notes.* Standard errors are in parentheses, ICC = Intraclass Correlation Coefficient, *** *p* < 0.001.

**Table 9:** Results for the hierarchical linear models predicting performance (ICC = 12%).

Parameters	Model 1	Model 2
Intercept	4.90 *** (0.05)	4.92 *** (0.04)
Level 1 (teams)		
Teamwork	−0.19 *** (0.04)	−0.19 *** (0.05)
Team size	−0.01 (0.00)	−0.01 (0.00)
Horizontal Trust	0.31 *** (0.05)	0.31 *** (0.05)
Level 2 (organizations)		
Organizational size		−0.00 (0.00)
Vertical Trust		0.04 (0.10)

*Notes.* Standard errors are in parentheses, ICC = Intraclass Correlation Coefficient, *** *p* < 0.001.

## Appendix A. Scale and Sources

**Variable**	**Items/Scales**	**Source** **Adapted from**	**Example of Item**
Healthy Organizational Practices	1. Work Family Balance 2. Mobbing Prevention 3. Psychosocial health 4. Organizational Communication	Salanova et al. (2012)	“In this company there are practices to facilitate the workers’ work–family balance”
Team Resources	1. Autonomy (task)	Jackson, Wall, Martin, and Davis (1993)	“In my work unit we decide when to begin, finish and the order in which we do the tasks”
	2. Feedback (task)	Jackson, Wall, Martin, and Davis (1993)	“The work we do gives us much information to know how well you are doing”
	3. Coordination 4. Supportive Team Climate	Salanova et al. (2012) Van Muijen et al. (1999)	“In my work unit we are coordinated with each other” “‘In my team, constructive criticism is rewarded’
Control Variable	5. Teamwork 6. Team size 7. Organizational size	Salanova et al. (2012)	“My work unit consists of people with appropriate and complementary expertise” Total number of members per team Total number of employees per organization
Organizational Trust	8. Vertical trust	Huff and Kelley (2003)	“In this company there is a high level of trust in management”
	9. Horizontal trust	McAllister (1995)	“In this company we can share our ideas, emotions and hopes”
Team Performance (rated by immediate supervisors) Organizational Performance	10. In-role performance	Goodman and Svyantek (1999)	“The team that I supervise performs all the functions and tasks demanded by the job”
	11. Extrarole performance 12. ROA	Goodman and Svyantek (1999) SABI database (http://sabi.bvdep.com)	“In the team that I supervise employees perform roles that are not formally required but which improve the organizational reputation” Dividing a company’s annual earnings by its total assets, obtaining a percentage

## References

[1] Mayer R.C., Davis J.H., Schoorman F.D. (1995). An Integrative Model of Organizational Trust. Acad. Manag. Rev..

[2] Falcone R., Colì E., Felletti S., Sapienza A., Castelfranchi C., Paglieri F. (2020). All We Need Is Trust: How the COVID-19 Outbreak Reconfigured Trust in Italian Public Institutions. Front. Psychol..

[3] Fulmer C.A., Gelfand M.J. (2011). At What Level (and in Whom) We Trust: Trust Across Multiple Organizational Levels. SSRN Electron. J..

[4] Wong Y.-T., Ngo H.-Y., Wong C.-S. (2003). Antecedents and Outcomes of Employees’ Trust in Chinese Joint Ventures. Asia Pac. J. Manag..

[5] Wöhrle J., van Oudenhoven J.P., Otten S., van der Zee K.I. (2015). Personality Characteristics and Workplace Trust of Majority and Minority Employees in the Netherlands. Eur. J. Work Organ. Psychol..

[6] Kramer R.M., Cook K.S. (2004). Trust and Distrust in Organizations: Dilemmas and Approaches.

[7] Wahda W., Mursalim M., Fauziah F., Asty A. (2020). Extra-Role Behavior Improvement Model: Organizational Learning Culture, Organizational Trust, and Organizational Justice Approach. Int. J. Eng. Bus. Manag..

[8] Dasgupta P., Gambetta D. (2000). Trust as a Commodity. Trust: Making and Breaking Cooperative Relations.

[9] Vokić N.P., Bilušić M.R., Najjar D. (2020). Building Organizational Trust through Internal Communication. Corp. Commun. Int. J..

[10] Bierstedt R., Blau P.M. (1964). Exchange and Power in Social Life.

[11] Acosta H., Salanova M., Llorens S. (2012). How Organizational Practices Predicted Team Work Engagement: The Role of Organizational Trust. Cienc. Trab..

[12] Dirks K.T., Ferrin D.L. (2002). Trust in Leadership: Meta-Analytic Findings and Implications for Research and Practice. J. Appl. Psychol..

[13] Kiffin-Petersen S., Cordery J. (2003). Trust, Individualism and Job Characteristics as Predictors of Employee Preference for Teamwork. Int. J. Hum. Resour. Manag..

[14] Walumbwa F.O., Luthans F., Avey J.B., Oke A. (2009). Retracted: Authentically Leading Groups: The Mediating Role of Collective Psychological Capital and Trust. J. Organ. Behav..

[15] Martínez-Tur V., Molina A., Moliner C., Gracia E., Andreu L., Bigne E., Luque O. (2019). Reciprocity of Trust Between Managers and Team Members. Pers. Rev..

[16] De Cremer D., van Dijke M., Schminke M., De Schutter L., Stouten J. (2018). The Trickle-Down Effects of Perceived Trustworthiness on Subordinate Performance. J. Appl. Psychol..

[17] Guinot J., Chiva R. (2019). Vertical Trust within Organizations and Performance: A Systematic Review. Hum. Resour. Dev. Rev..

[18] Klein K.J., Dansereau F., Hall R.J. (1994). Levels Issues in Theory Development Data Collection, and Analysis. Acad. Manag. Rev..

[19] Rousseau D.M., Sitkin S.B., Burt R.S., Camerer C. (1998). Not So Different After All: A Cross-Discipline View of Trust. Acad. Manag. Rev..

[20] Colquitt J.A., Scott B.A., LePine J.A. (2007). Trust, Trustworthiness, and Trust Propensity: A Meta-Analytic Test of Their Unique Relationships with Risk Taking and Job Performance. J. Appl. Psychol..

[21] Creed D.R., Miles R.E., Kramer R.M., Tyler T.R. (1996). Trust in Organizations: A Conceptual Framework Linking Organizational Forms, Managerial Philosophies, and the Opportunity Costs of Controls. Trust in Organizations: Frontiers of Theory and Research.

[22] Legood A., Thomas G., Sacramento C. (2016). Leader Trustworthy Behavior and Organizational Trust: The Role of the Immediate Manager for Cultivating Trust. J. Appl. Soc. Psychol..

[23] Salanova M., Llorens S., Cifre E., Martínez I.M.M. (2012). We Need a Hero! Toward a Validation of the Healthy and Resilient Organization (HERO) Model. Group Organ. Manag..

[24] Costa A.C. (2003). Work Team Trust and Effectiveness. Pers. Rev..

[25] Frazier M.L., Gooty J., Little L.M., Nelson D.L. (2015). Employee Attachment: Implications for Supervisor Trustworthiness and Trust. J. Bus. Psychol..

[26] Wright P.M., McMahan C.G. (1992). Theoretical Perspectives for Strategic Human Resource Management. J. Manag..

[27] Cano C.R., Bruhn J.G. (2004). Trust and the Health of Organizations. Acad. Manag. Rev..

[28] Tremblay M., Cloutier J., Simard G., Chênevert D., Vandenberghe C. (2010). The Role of HRM Practices, Procedural Justice, Organizational Support and Trust in Organizational Commitment and in Role and Extra-Role Performance. Int. J. Hum. Resour. Manag..

[29] Bacon N., Hoque K. (2005). HRM in the SME Sector: Valuable Employees and Coercitive Networks. Hum. Resour. Manag..

[30] Schneider B., Hanges P.J., Smith D.B., Salvaggio A.N. (2003). Which Comes First: Employee Attitudes or Organizational Financial and Market Performance?. J. Appl. Psychol..

[31] Carlsen A. (2008). Positive Dramas: Enacting Self-Adventures in Organizations. J. Posit. Psychol..

[32] Fredrickson B., Dutton J. (2008). Unpacking Positive Organizing: Organizations as Sites of Individual and Group Flourishing. J. Posit. Psychol..

[33] Kath L.M., Magley V.J., Marmet M. (2010). The Role of Organizational Trust in Safety Climate’s Influence on Organizational Outcomes. Accid. Anal. Prev..

[34] Pirson M., Malhotra D. (2011). Foundations of Organizational Trust: What Matters to Different Stakeholders?. Organ. Sci..

[35] Xanthopoulou D., Bakker A.B., Demerouti E., Schaufeli W.B. (2009). Work Engagement and Financial Returns: A Diary Study on the Role of Job and Personal Resources. J. Occup. Organ. Psychol..

[36] Wells C.V., Kipnis D. (2001). Trust, Dependency, and Control in the Contemporary Organization. J. Bus. Psychol..

[37] Saunders M.N., Dietz G., Thornhill A. (2014). Trust and Distrust: Polar Opposites, or Independent but Co-existing?. Hum. Relat..

[38] Lyubomirsky S., King L., Diener E. (2005). The Benefits of Frequent Positive Affect: Does Happiness Lead to Success?. Psychol. Bull..

[39] Hakanen J.J., Bakker A.B., Schaufeli W.B. (2006). Burnout and Work Engagement among Teachers. J. Sch. Psychol..

[40] Harms P., Bai Y., Han G.H. (2016). How Leader and Follower Attachment Styles are mediated by Trust. Hum. Relat..

[41] Langfred C.W. (2004). Too Much of a Good Thing? Negative Effects of High Trust and Individual Autonomy in Self-Managing Teams. Acad. Manag. J..

[42] Peñalver J., Salanova M., Martínez I.M., Schaufeli W. (2019). Happy-Productive Groups: How Positive Affect Links to Performance through Social Resources. J. Posit. Psychol..

[43] Whitman D.S., Van Rooy D.L., Viswesvaran C. (2010). Satisfaction, Citizenship Behaviors, and Performance in Work Units: A Meta-Analysis of Collective Construct Relations. Pers. Psychol..

[44] Guzzo R.F., Wang X., Madera J.M., Abbott J. (2021). Organizational Trust in Times of COVID-19: Hospitality Employees’ Affective Responses to Managers’ Communication. Int. J. Hosp. Manag..

[45] Kozlowski S.W.J., Bell B.S., Borman W.C., IIgen D.R., Kimoski R.J. (2003). Work Group and Teams in Organizations. Handbook of Psychology: Industrial and Organizational Psychology.

[46] Devine D.J., Fogel M.H., Philips J.L. (2001). Do Smarter Teams Really Do Better: A Review and Meta-Analysis of Cognitive Ability and Team Performance. Small Group Res..

[47] Goodman S.A., Svyantek D.J. (1999). Person–Organization Fit and Contextual Performance: Do Shared Values Matter. J. Vocat. Behav..

[48] Mishra A., Kramer M., Tyler T.R. (1996). Organizational Responses to Crisis: The Centrality of Trust. Trust in Organizations: Frontiers of Theory and Research.

[49] Bijlsma K., van de Bunt G.G. (2003). Antecedents of Trust in Managers: A “Bottom Up” Approach. Pers. Rev..

[50] Ferres N., Connell J., Travaglione A. (2004). Co-Worker Trust as a Social Catalyst for Constructive Employee Attitudes. J. Manag. Psychol..

[51] Vanhala M., Heilmann P., Salminen H. (2016). Organizational Trust Dimensions as Antecedents of Organizational Commitment. Knowl. Process. Manag..

[52] Curado C., Vieira S. (2019). Trust, Knowledge Sharing and Organizational Commitment in SMEs. Pers. Rev..

[53] De Jong B.A., Dirks K.T., Gillespie N. (2016). Trust and Team Performance: A Meta-Analysis of Main Effects, Moderators, and Covariates. J. Appl. Psychol..

[54] Nunnally J.C., Bernstein I.H. (1994). Psychometric Theory.

[55] Huff L., Kelley L. (2003). Levels of Organizational Trust in Individualist versus Collectivist Societies: A Seven-Nation Study. Organ. Sci..

[56] McAllister D.J. (1995). Affect and Cognition Based Trust as Foundations for Interpersonal Cooperation in Organizations. Acad. Manag. J..

[57] Brewer M.B., Kramer R.M. (1986). Choice Behavior in Social Dilemmas: Effects of Social Identity, Group Size, and Decision Framing. J. Personal. Soc. Psychol..

[58] Le Blanc P.M., González-Romá V. (2012). A Team Level Investigation of the Relationship between Leader–Member Exchange (LMX) Differentiation, and Commitment and Performance. Leadersh. Q..

[59] Lebreton J.M., Senter J.L. (2008). Answers to 20 Questions about Interrater Reliability and Interrater Agreement. Organ. Res. Methods.

[60] Murphy K.R., Myors B. (1998). Statistical Power Analysis: A Simple and General Model for Traditional and Modern Hypothesis Test.

[61] Burke M.J., Finkelstein L.M., Dusig M.S. (1999). On Average Deviation Indices for Estimating Interrater Agreement. Organ. Res. Methods.

[62] Podsakoff P.M., MacKenzie S.M., Lee J., Podsakoff N.P. (2003). Common Method Variance in Behavioral Research: A Critical Review of the Literature and Recommended Remedies. J. Appl. Psychol..

[63] Preacher K.J., Hayes A.F. (2004). SPSS and SAS Procedures for Estimating Indirect Effects in Simple Mediation Models. Behav. Res. Methods, Instrum. Comput..

[64] Shrout P.E., Bolger N. (2002). Mediation in Experimental and Non-Experimental Studies: New Procedures and Recommendations. Psychol. Methods.

[65] Efron B., Tibshirani R. (1993). An Introduction to the Bootstrap.

[66] Arbuckle J.L. (1997). Amos Users’ Guide Version 4.0..

[67] Bentler P.M. (1990). Comparative Fit Indexes in Structural Equation Models. Psychol. Bull..

[68] Browne M.W., Cudeck R. (1993). Alternative Ways of Assessing Model Fit. Testing Structural Equation Models.

[69] Hu L.T., Bentler P.M. (1999). Cut-Off Criteria for Fit Indices in Covariance Structure Analysis: Conventional Criteria Versus New Alternatives. Struct. Equ. Model..

[70] Baron R.M., Kenny D.A. (1986). The Moderator-Mediator Variable Distinction in Social Psychological Research: Conceptual, Strategicand Statistical Consideration. J. Personal. Soc. Psychol..

[71] Sobel M.E., Van Der Heijden P.G.M., Van Gils G., Bouts J., Hox J.J. (1987). Direct and Indirect Effects in Linear Structural Equation Models. Sociol. Methods Res..

[72] Gavin M.B., Hofmann D.A. (2002). Using Hierarchical Linear Modeling to Investigate the Moderating Influence of Leadership Climate. Leadersh. Q..

[73] Bliese P., Klein K., Kozlowski S. (2000). Within-Group Agreement, Non-Independence, and Reliability. Implications for Data Analysis. Multilevel Theory, Research, and Methods in Organizations: Foundations, Extensions and New Directions.

[74] Hox J. (2010). Multilevel Analyses: Techniques and Applications.

[75] Jöreskog K.G., Sörbom D. (2006). LISREL 8.80 for Windows [Computer Software].

[76] Hofmann D.A., Griffin M.A., Gavin M.B., Klein K., Kozlowski S. (2000). The Application of Hierarchical Linear Modeling to Organizational Research. Multi Level Theory, Research, and Methods in Organizations: Foundations, Extensions, and New Directions.

[77] Covey S.M.R. (2006). Speed of Trust. The One Thing that Change Everything.

[78] Kim B.-J. (2019). Unstable Jobs Cannot Cultivate Good Organizational Citizens: The Sequential Mediating Role of Organizational Trust and Identification. Int. J. Environ. Res. Public Health.

[79] Andersen J.A. (2005). Trust in Managers: A Study of Why Swedish Subordinates Trust their Managers. Bus. Ethic. A Eur. Rev..

[80] Torrente P., Salanova S., Llorens S., Schaufeli W.B. (2012). Teams Make it Work: How Teamwork Engagement Mediates between Social Resources and Performance in Teams. Psicothema.

